# The Effect of Buffer Types on the In_0.82_Ga_0.18_As Epitaxial Layer Grown on an InP (100) Substrate

**DOI:** 10.3390/ma11060975

**Published:** 2018-06-08

**Authors:** Min Zhang, Zuoxing Guo, Liang Zhao, Shen Yang, Lei Zhao

**Affiliations:** 1 Key Laboratory of Automobile Materials (Ministry of Education), Jilin University, Changchun 130025, China; zhangmin16@mails.jlu.edu.cn (M.Z.); guozx@jlu.edu.cn (Z.G.); 2 College of Materials Science and Engineering, Jilin University, Nanling Campus, Changchun 130025, China; zhaoliang14@mails.jlu.edu.cn (L.Z.); yangshen16@mails.jlu.edu.cn (S.Y.)

**Keywords:** In_0.82_Ga_0.18_As/InP, single buffer layer, graded buffer, superlattice buffer

## Abstract

In_0.82_Ga_0.18_As epitaxial layers were grown on InP (100) substrates at 530 °C by a low-pressure metalorganic chemical vapor deposition (LP-MOCVD) technique. The effects of different buffer structures, such as a single buffer layer, compositionally graded buffer layers, and superlattice buffer layers, on the crystalline quality and property were investigated. Double-crystal X-ray diffraction (DC-XRD) measurement, Raman scattering spectrum, and Hall measurements were used to evaluate the crystalline quality and electrical property. Scanning electron microscope (SEM), atomic force microscope (AFM), and transmission electron microscope (TEM) were used to characterize the surface morphology and microstructure, respectively. Compared with the In_0.82_Ga_0.18_As epitaxial layer directly grown on an InP substrate, the quality of the sample is not obviously improved by using a single In_0.82_Ga_0.18_As buffer layer. By introducing the graded In_x_Ga_1−x_As buffer layers, it was found that the dislocation density in the epitaxial layer significantly decreased and the surface quality improved remarkably. In addition, the number of dislocations in the epitaxial layer greatly decreased under the combined action of multi-potential wells and potential barriers by the introduction of a In_0.82_Ga_0.18_As/In_0.82_Al_0.18_As superlattice buffer. However, the surface subsequently roughened, which may be explained by surface undulation.

## 1. Introduction

In_x_Ga_1−x_As (0 < x < 1)—known as ternary compound semiconductor materials—can well cover the infrared radiation of 1~3 μm. It is a direct band gap semiconductor of the III–V family with a sphalerite structure, making it a suitable choice for creating short-wavelength infrared detectors [1,2,3,4]. Because of its excellent characteristics and mature preparation technology, In_x_Ga_1−x_As materials have attracted much research attention and have been widely used in the fields of civil, military, space remote sensing, spectroscopy, and so on [5,6,7,8]. The future is bright for structures and devices based on InP material systems because of their higher integration, higher frequency, higher power, higher power efficiency, lower noise, and lower cost. In_0.53_Ga_0.47_As epitaxial material covering a wavelength of 0.9–1.7 μm is lattice-matched to an InP substrate, which has been developed mainly for fiber communication applications. However, as there is no lattice-matched substrate for In_0.82_Ga_0.18_As materials, the large lattice mismatch between the In_0.82_Ga_0.18_As epitaxial layer and the substrate will result in misfit dislocations or other defects, which will destroy the material quality and further weaken the performance of devices [9]. In the strain relaxation process of epitaxial growth, threading dislocations (TDs) are concomitantly generated with misfit dislocations (MDs), which are also detrimental to optoelectronic devices. Therefore, an additional buffer layer is a feasible method commonly used to solve the problem of a large lattice mismatch which has always been a serious problem limiting the appliance of semiconductor hererostructures. Many efforts using low-pressure metalorganic chemical vapor deposition [10], molecular beam epitaxy [11], or low-pressure metalorganic vapor phase epitaxy [12] with different types of buffer structures have been made, thus the qualities of InGaAs materials have been effectively evaluated. In previous reports, various buffer structures, which were implemented to reduce the dislocation density, have been explored, including a thick uniform buffer [13], a compositionally linearly-graded or step-graded buffer [14,15,16,17], and a digitally graded buffer [18]. For example, an In_0.8_Ga_0.2_As buffer layer was introduced between an In_0.8_Ga_0.2_As epitaxial layer and an InP (100) substrate and showed that the heterostructure with a buffer thickness of 100 nm had the optimum properties [10]. In addition, the wavelength extended In_0.8_Ga_0.2_As photodiodes grown on InP substrates were investigated by using a linearly graded In_x_Al_1−x_As buffer and an excellent performance was demonstrated [19]. Moreover, there is another buffer method known as the superlattice buffer. A superlattice is an artificial crystal consisting of two or more materials with periodic and alternate growth which has been used in the making of quantum cascade lasers, superlattice nanowires, and so on [20,21,22,23]. Zhao Xiaomeng et al. inserted the InSb/In_0.9_Al_0.1_Sb superlattice between InSb thin films and a GaAs (001) substrate by the molecular beam epitaxy (MBE), and deduced that the superlattice (SL) structure could effectively prevent dislocation propagating to the upper InSb thin films [24]. Obviously, the introduction of superlattice layers between the epitaxial layer and substrate has also shown some better possibilities to reduce the dislocations [25,26]. 

Hence, a comprehensive understanding of dislocation-mediated strain relaxation during III–V heteroepitaxial growth is very necessary. One purpose of this article is to compare and evaluate the effect of different buffer structures, such as a single buffer layer, compositionally graded buffer layers, and superlattice buffer layers, on the crystalline quality, surface morphology, and structure of an In_0.82_Ga_0.18_As epitaxial layer grown on an InP (100) substrate by a low-pressure metalorganic chemical vapor deposition (LP-MOCVD) technique. The mechanism of reducing dislocations is also explored. 

## 2. Experiments

In_0.82_Ga_0.18_As epitaxial layers were grown on semi-insulating InP (100) substrates by a low pressure metalorganic chemical vapor deposition technique. Trimethylindium (TMIn), trimethylgallium (TMGa), and 10% arsine (AsH_3_) in H_2_ were used as group III and V source materials, respectively. Phosphine (PH_3_) was employed as a protective atmosphere. Prior to the growth, the InP (100) substrates on the graphite susceptor were heated at 630 °C for 10 min to remove the oxide layer from the surface using inductively coupled radio frequency power in the phosphine atmosphere. The reactor pressure was maintained at 1 × 10^4^ Pa. When the temperature in the reaction chamber fell to 530 °C, the growth of the heterostructure for sample A was carried out and was directly grown on the InP (100) substrate. For sample B, when the temperature of the reaction chamber was reduced to 450 °C, the 100 nm thick In_0.82_Ga_0.18_As buffer layer was grown on the InP (100) substrate and then the In_0.82_Ga_0.18_As epitaxial layer was grown when the temperature rose to 530 °C. For sample C and sample D, the two heterostructures were grown with different buffer structures at 530 °C. Sample C was grown with In_x_Ga_1−x_As buffer layers that In composition x graded from 0.53, 0.62, 0.72, to 0.82 in which every layer of the structure was 25 nm; sample D with a 9-pair In_0.82_Ga_0.18_As/In_0.82_Al_0.18_As superlattice where the thickness ratio between the In_0.82_Ga_0.18_As layer and In_0.82_Al_0.18_As layer was fixed to 1:1 and the total thickness of one pair was 10 nm, in addition to a 25 nm thick In_0.53_Al_0.47_As layer inserted between the InP substrate and superlattice which was to ensure a smooth surface for the subsequent layer growth. In these samples, all the In_0.82_Ga_0.18_As epitaxial layers were also grown at 530 °C by LP-MOCVD with a thickness of 1500 nm. 

The surface morphologies were evidenced and analyzed by atomic force microscopy (AFM, Multimode 8, Bruker, Billerica, MA, USA) and scanning electron microscope (SEM, VEGA 3, Tescan, Brno, Czech Republic). Double-crystal X-ray diffraction (DC-XRD) and Raman scattering measurements (UV-Horiba, Horiba, Tokyo, Japan) were used to investigate the composition and crystal quality of the epitaxial layers. Transmission electron microscopy (TEM, JEM-2100F, JEOL, Toyko, Japan) was performed to examine the microstructure and the distribution of dislocations in the In_0.82_Ga_0.18_As/InP heterostructures. Hall measurements (Lake-7704A, Lake, Lower Lake, CA, USA) were carried out to characterize and evaluate the properties of the samples.

## 3. Results and Discussions

**Figure 1:**
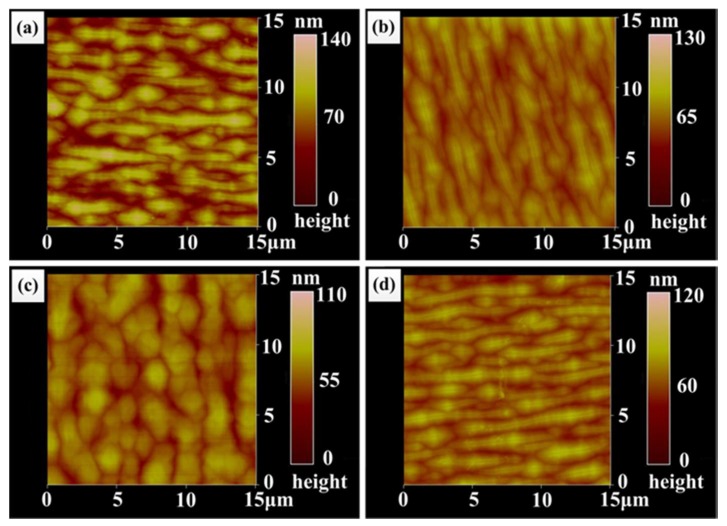
Atomic force microscope (AFM) images of the In_0.82_Ga_0.18_As epitaxial layers of four samples: (**a**) sample A; (**b**) sample B; (**c**) sample C and (**d**) sample D.

AFM scans over 15 × 15 μm^2^ were performed to investigate the surface morphology of the In_0.82_Ga_0.18_As epitaxial layers of four samples. Slightly disordered corrugation patterns were observed, as shown in Figure 1a–d, which may be associated with the three-dimensional growth mode of the epitaxial layer [27]. It can be clearly seen that the surface morphologies of sample A, B, and D (Figure 1a,b,d) are very similar in terms of the patterns of narrow and longer corrugations. However, the morphology in sample C (Figure 1c) is distinct, consisting of broadened corrugations. Root-mean-square (RMS) roughness and height difference of the four samples are summarized in Table 1. Combined with Table 1, it is clear that the RMS values of the four samples decrease at first and then increase slightly; the change in height differences has the same trend. Sample A without buffer structure has maximum values of 26.7 nm and 80.4 nm for surface roughness and height difference, respectively. However, sample C with graded buffer layers has minimum values of 15.4 nm and 48.6 nm for surface roughness and height difference, respectively. According to Vegard’s law, the calculated lattice constant of In_0.82_Ga_0.18_As materials is 5.985 Å, and thus the lattice mismatch between In_0.82_Ga_0.18_As and the InP substrate is 2%. For sample A, the In_0.82_Ga_0.18_As epitaxial layer directly grown on the InP substrate was subjected to the action of the larger mismatched strain which brought lots of defects into the epitaxial layer, resulting in a poorer surface quality. However, various buffer structures in samples B, C, and D reduced the defects in the epitaxial layer, so the surface qualities of sample B–D were improved by the introduction of a buffer layer; these surface qualities being better than that of sample A.

**Figure 2:**
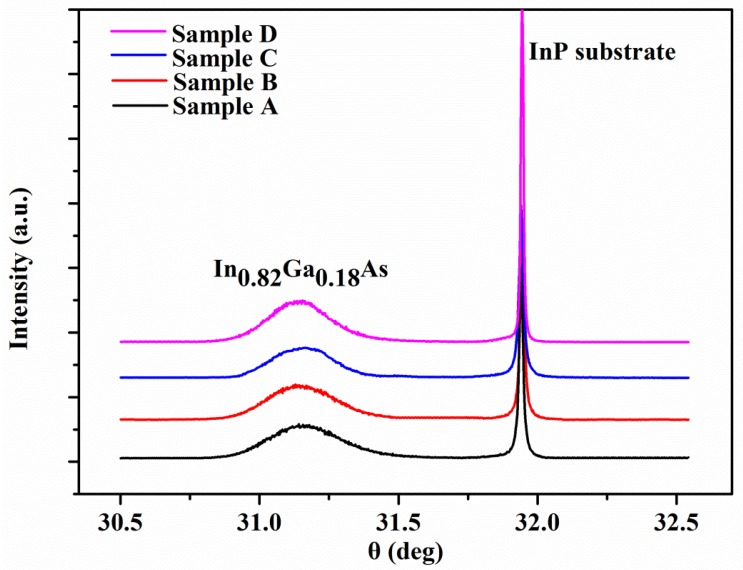
The double-crystal X-ray diffraction (DC-XRD) ω scans of In_0.82_Ga_0.18_As epitaxial layers grown on InP (100) substrates for sample A–D.

Figure 2 shows the double-crystal X-ray diffraction (DC-XRD) patterns for samples A–D. The two diffraction peaks corresponding to the In_0.82_Ga_0.18_As epitaxial layer and InP substrate, respectively, are clearly observed in Figure 2. Except for the In_0.82_Ga_0.18_As epitaxial layer and the InP (100) substrate, no other diffraction peaks were detected, which confirms that the In_0.82_Ga_0.18_As epitaxial layer has an uniform (100) rientation. According to the DC-XRD curves, the full width at half maximum (FWHM) values of the In_0.82_Ga_0.18_As epitaxial layer are calculated and summarized in Table 1. It is clear that the FWHM values decrease gradually from sample A to D, indicating that the crystal quality becomes better. The dislocation density of the four samples has the following formula:

(1) $$N_{\mathrm{dis}}=\frac{{\left(\mathrm{FWHM}\right)}^{2}}{{9b}^{2}},$$

where b is Burgers vector of materials and $N_{\mathrm{dis}}$ is dislocation density [28]. The dislocation densities calculated by the full width at half maximum (FWHM) method are also collected in Table 1. It was found that from sample A to D, the dislocation densities reduced, confirming the conclusion that the quality of crystallization improved. 

**Figure 3:**
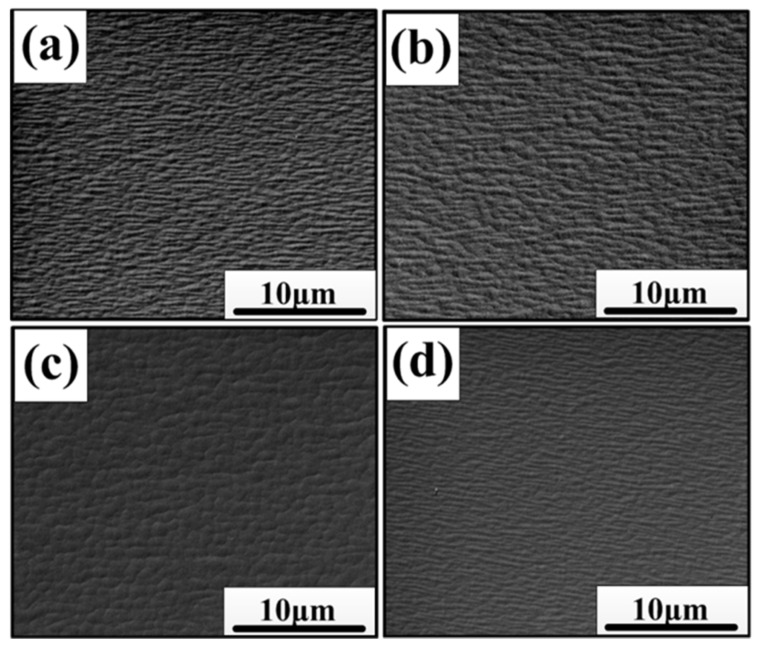
Scanning electron microscope (SEM) images of In_0.82_Ga_0.18_As epitaxial layers of the four samples: (**a**) sample A; (**b**) sample B; (**c**) sample C and (**d**) sample D.

The surface morphology of the In_0.82_Ga_0.18_As epitaxial layer was also examined by the scanning electron microscope. Figure 3a–d shows the surface morphologies of the In_0.82_Ga_0.18_As epitaxial layers of the four samples. It was found that the mounds elongated to form long ridges on the surface for sample A, sample B, and sample D, indicative of the misfit dislocation array at the interface [29]. For sample D, it was relatively flat when compared to the other three samples. Especially for sample C, the surface morphology was different, without pronounced heave, and became dominated by rumpled cross-hatching. Sample A and sample B grew with rougher three-dimensional (3D) features, many dislocations in the epitaxial layer (including the near surface) could have acted as channels for atomic diffusion, resulting in the different velocity of atom diffusion during the growth process, thus forming a rough surface with poor quality. In sample B, the single buffer layer had less ability to stop the dislocation with the slow relaxation of strain, so the surface quality of sample B did not obviously improve, as shown in Figure 3b. In sample D, the dislocation density decreased significantly so the large 3D features were weakened (Figure 3d), forming a flatter surface. For sample C with the graded buffer layers, as the stress relaxation changed into a rapid relaxation mechanism, the driving force of coarsening was weakened, and so the rough three-dimensional features disappeared. Moreover, because of the lower dislocation density, the atomic diffusion rate of the surface was uniform. So there was no pronounced heave on the surface forming the cross-hatching, as shown in Figure 3c.

**Figure 4:**
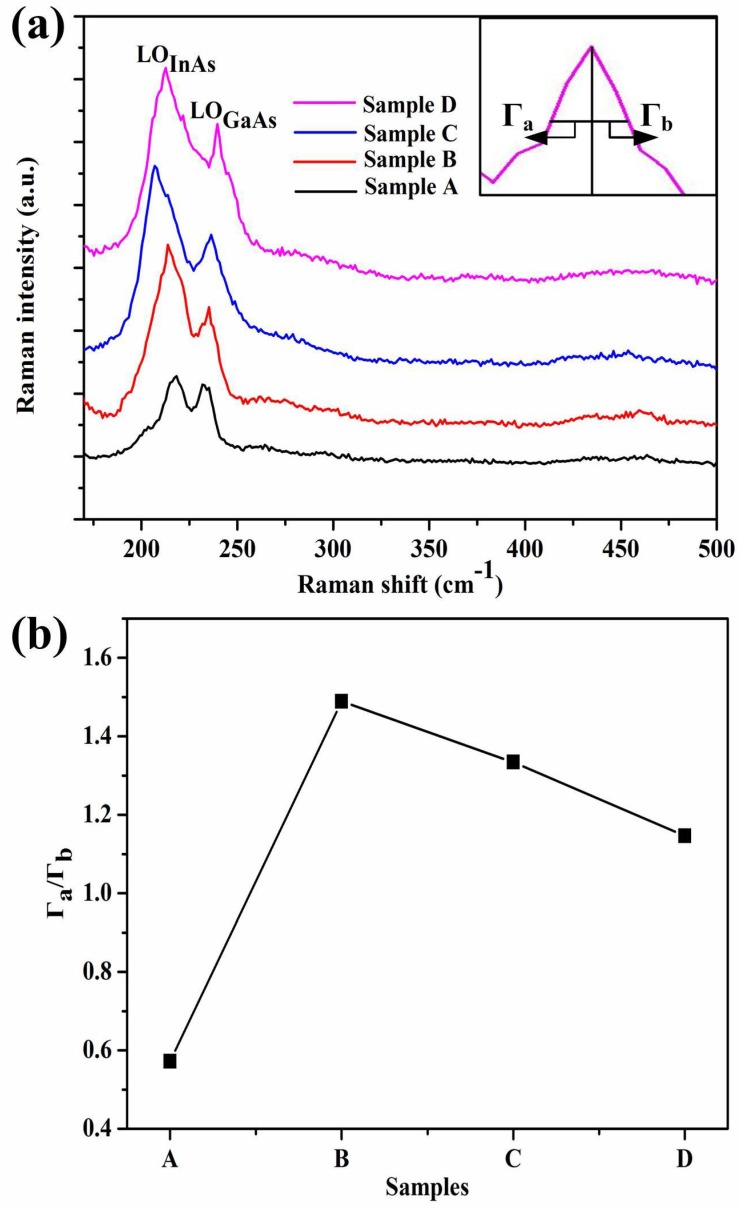
(**a**) Raman spectra of In_0.82_Ga_0.18_As epitaxial layers grown on InP (100) substrates for samples A–D. The inset shows Γ_a_ and Γ_b_ which is used in the asymmetric ratio (Γ_a_/Γ_b_) of Raman scattering spectra; (**b**) line chart of the variation of asymmetric ratio (Γ_a_/Γ_b_) for samples A–D.

In addition to DC-XRD measurement, Raman scattering is an indirect way to characterize the crystalline quality of materials. Figure 4 shows the Raman spectra of In_0.82_Ga_0.18_As epitaxial layers for samples A–D. Two-mode Raman peaks are observed in each spectrum which correspond to longitudinal optical (LO) phonon modes of InAs and GaAs, respectively [30]. Because of the high content of indium composition, the peak corresponding to InAs-like LO is strong and sharp. In this experiment, the back scattering of (100) crystal orientation was adopted. According to the scattering selection rule of zinc-blend-type structure, there are only LO phonon mode peaks under the (100) backscattering in the In_0.82_Ga_0.18_As epitaxial layer. Furthermore, the asymmetry ratio (Γ_a_/Γ_b_) of Raman scattering spectra can be used to characterize the crystalline quality of samples [31]. The closer the asymmetric ratio is to 1, the higher the quality of the material. For In_0.82_Ga_0.18_As materials, the asymmetric ratios (Γ_a_/Γ_b_) of GaAs-like LO-phonon peaks are selected to characterize the crystalline quality of samples, which is displayed in the inset of Figure 4a. The calculated values of the asymmetric ratios of samples A–D are summarized in Figure 4b. It can be seen that the asymmetric ratios of samples A–D are not all 1, and the asymmetric ratio of the samples is closer to 1 increasingly from sample A to sample D, indicating that the crystalline quality for samples A–D becomes better and better. The results show that the crystalline quality of In_0.82_Ga_0.18_As epitaxial layers can be improved by the introduction of buffer layers with different structures, which is in accord with the DC-XRD measurements.

**Figure 5:**
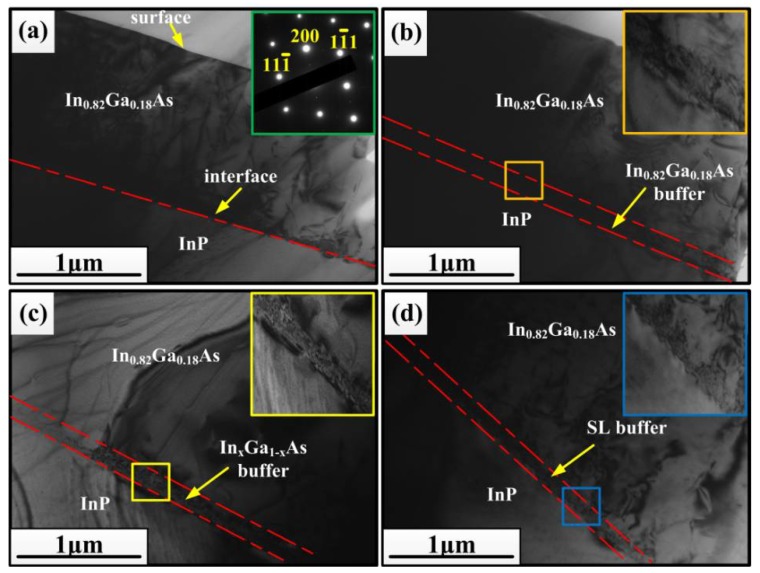
The transmission electron microscopy (TEM) images of In_0.82_Ga_0.18_As epitaxial layer grown on InP (100) substrates: (**a**) sample A without buffer layer; (**b**) sample B with In_0.82_Ga_0.18_As buffer layer; (**c**) sample C with graded In_x_Ga_1−x_As buffer layers; (**d**) sample D with In_0.82_Ga_0.18_As/In_0.82_Al_0.18_As superlattice (SL) buffer; the insets are the magnified images of three small frames in (**b**,**c**,**d**).

Figure 5 shows the TEM images of the four In_0.82_Ga_0.18_As/InP heterostructures with or without buffer structures. As is well known, the variation of microstructure can affect the quality of materials to a great extent. Because of the large lattice mismatch of about 2% and a_e_ > a_s_ (a_e_ and a_s_ denote the lattice parameter of the epitaxial layer and the substrate, respectively), the In_0.82_Ga_0.18_As epitaxial layer of sample A is a positive mismatch to the InP substrate and is under compression strain. It can be seen in Figure 5a that a large number of dislocations were generated at the interface between the epitaxial layer and substrate, forming a dark, indistinguishable area. Many of the misfit dislocations interacted with each other and formed misfit dislocations networks in order to release the large compressive strain. During growth process, there are many dislocations stretching upward and even propagating to the surface, which causes aggregation of the surface atoms, affects the surface structure of the epitaxial layer, and finally forms a very rough surface, as shown in Figure 3a. For sample B, the 100 nm thick In_0.82_Ga_0.18_As buffer layer was inserted between the epitaxial layer and InP substrate. The In_0.82_Ga_0.18_As buffer layer grown on the substrate was in favor of the growth of the In_0.82_G_a0.18_As epitaxial layer because they matched well with each other. Furthermore, both the growth of the buffer layer and epitaxial layer are a positive mismatch to the InP substrate. By comparing this with previous reports performed by Zhao Liang et al. [32] and Wei Qiulin et al. [33] whereby the low temperature growth (450 °C) of the In_0.82_Ga_0.18_As buffer layer provides a nucleation center with the same substrate orientation, which acts like a template of the epitaxial layer and provides a nuclear surface for the subsequent growth for In_0.82_Ga_0.18_As epitaxial layer. When the growth of the buffer layer is completed, the temperature of the reaction chamber is gradually increased to the growth temperature of the epitaxial layer (530 °C), which is a process of high temperature annealing for the buffer layer. Then, the surface of the buffer layer is recrystallized, which makes the surface of the buffer layer smooth and can effectively release the thermal strain caused by the mismatch of coefficient of thermal expansion between In_0.82_Ga_0.18_As and InP. It can clearly be seen in Figure 5b that although the part of the dislocations is confined to the buffer layer, there are still many dislocations propagating into the epitaxial layer and spreading near the surface. Maybe it is because the thickness of the buffer layer cannot compensate the misfit strain between the In_0.82_Ga_0.18_As epitaxial layer and InP substrate and accommodate the dislocations induced by the strain, and the high In content buffer layer produce more dislocations which might extend to the epitaxial layers. Therefore, the differences in the surface morphology and roughness are very small for sample A and sample B, as listed in Table 1 and shown in Figure 3a,b. For sample C, the In content of In_x_Ga_1−x_As buffer layers inserted between the top In_0.82_Ga_0.18_As epitaxial layer and InP substrate grades from 0.53 to 0.82, in which the first layer with an In content of 0.53 and the last layer with an In content of 0.82 match well with the InP substrate and In_0.82_Ga_0.18_As epitaxial layer, respectively. Furthermore, every layer (except for the first layer) in the graded buffer and the top In_0.82_Ga_0.18_As epitaxial layer are positive mismatching to the InP substrate and under compression strain. At the same time, the thicknesses of the In_x_Ga_1−x_As buffer layers of each component are larger than the critical thickness, so a large number of dislocations and defects are restricted in the buffer layers. The graded buffer layers give play to three functions: releasing stress, restraining surface undulation, and restricting dislocations. It can be clearly seen from Figure 5c that the dislocations are mainly distributed at the interior of the graded buffer layers. The stress between every layer in the graded buffer structure would make the misfit dislocations bend near the internal interfaces, and thus there are few misfit dislocations propagating through the structure and further spreading to the surface, as shown in Figure 3c, forming a very smooth surface. By using the graded buffer layers, the mismatched dislocations are distributed at different heights of the graded buffer structure, instead of being confined to a single buffer layer, which provides a larger amount of space for dislocation movement and reduces the probability of mutual pinning, thus effectively reducing the penetration dislocation density in the epitaxial growth. As regards reducing the dislocation density, the greatest advantage of utilizing the graded In_x_Ga_1−x_As buffer layer is that the dislocations annihilation cannot only occur between different dislocations, but can also occur between different layers, which gives much more possibilities for dislocation annihilation. Therefore, the dislocation density of sample C reduced significantly and the surface quality improved remarkably, as listed in Table 1. In sample D, the In_0.82_Ga_0.18_As/In_0.82_Al_0.18_As superlattice was employed as buffer to reduce the dislocation density. For In_0.82_Ga_0.18_As/In_0.82_Al_0.18_As superlattice structure, In_0.82_Ga_0.18_As layers and In_0.82_Al_0.18_As layers form alternate potential wells and potential barriers, respectively. On the one hand, the defects such as dislocations are limited in the In_0.82_Ga_0.18_As potential wells and have a lower energy state, thus In_0.82_Ga_0.18_As layers play a trapping role in the structure. On the other hand, since the defects already have a lower energy state in the potential wells, they do not possess enough energy to pass through the barriers, therefore the barriers formed by In_0.82_Al_0.18_As layers have a blocking effect [34,35]. It can be seen from Figure 5d that a high density of dislocations is confined in the In_0.82_Ga_0.18_As/In_0.82_Al_0.18_As superlattice buffer area under the combined action of multi-potential wells and potential barriers. Although there are still a few dislocations passing through the buffer area and extending to the In_0.82_Ga_0.18_As epitaxial layer, the number of dislocations reduced in the epitaxial layer and these dislocations did not propagate to the surface. Therefore, the In_0.82_Ga_0.18_As/In_0.82_Al_0.18_As superlattice buffer area can effectively play the role of “dislocation filter”. However, the elongated mounds forming long ridges appear again on the surface of sample D (Figure 3d), similar to sample A. As shown in Table 1, by comparing with sample C, the surface roughness of sample D also slightly increases. This phenomenon may be explained by the other strain relaxation mechanism, known as surface undulation [36]. While a large misfit strain could not release enough by dislocation arrays, surface undulation will relax part of the strain but increase the surface roughness.

**Figure 6:**
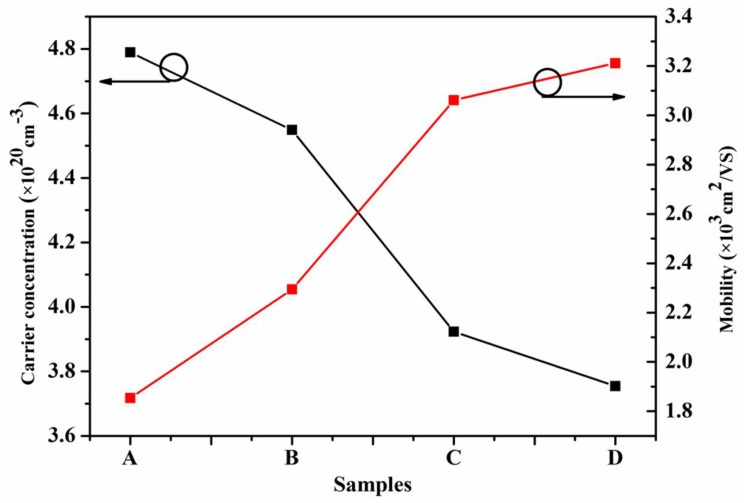
Variation of the mobility and carrier concentration of the In_0.82_Ga_0.18_As epitaxial layers grown on InP (100) substrates.

In addition, the quality of the In_0.82_Ga_0.18_As epitaxial layers is further characterized by Hall measurements with a magnetic field of 7000 G at 300 K. The results of Hall measurements are shown in Figure 6. In the epitaxial layers, defects such as dislocations can serve as scattering centers for carriers and thus limit the mobility. Therefore, the crystalline quality and electron property of the epitaxial layers can be illustrated by the Hall results. It is clearly shown in Figure 6 that the introduction of various buffer structures between epitaxial layers and InP substrates has a strong influence on the carrier concentration and Hall mobility of the In_0.82_Ga_0.18_As epitaxial layers. From sample A to sample D, the measured carrier concentration reduced and the measured mobility increased. Sample D with graded superlattice buffer layers has the lowest carrier concentration value (3.73 × 10^20^ cm^−3^) and the highest Hall mobility value (3.21 × 10^3^ cm^2^/VS). Meanwhile, the calculated dislocation density in the In_0.82_Ga_0.18_As epitaxial layer of sample D is the least. Therefore, sample D has the best quality in epitaxial growth. Therefore, it can be concluded that the higher the mobility and the lower the carrier concentration of the material, the better the quality of the epitaxial layer. 

## 4. Conclusions

In this work, In_0.82_Ga_0.18_As epitaxial layers were grown on InP (100) substrates by a low pressure metalorganic chemical vapor deposition (LP-MOCVD) technique. The effect of different buffer structures, such as a single buffer layer, compositionally graded buffer layers, and superlattice buffer layers, on the crystalline quality and surface morphology was investigated. When the In_0.82_Ga_0.18_As epitaxial layer was directly grown on the InP substrate, a large number of dislocations were generated and extended to the surface, resulting in a particularly poor quality sample. Using a single In_0.82_Ga_0.18_As buffer layer, the dislocation density reduced slightly. However, there were still some dislocations extending to the epitaxial layer and even propagating to the surface This was because the misfit strain between the In_0.82_Ga_0.18_As epitaxial layer and InP substrate could not compensate the single buffer layer. By introducing the graded In_x_Ga_1−x_As buffer layer, it was be shown that the dislocations were mainly distributed at the interior of the graded buffer layers, the dislocation density reduced significantly and the surface quality improved remarkably. The In_0.82_Ga_0.18_As/In_0.82_Al_0.18_As superlattice buffer can effectively play the role of "dislocation filter". The number of dislocations in the epitaxial layer greatly decreased under the combined action of multi-potential wells and potential barriers of the In_0.82_Ga_0.18_As/In_0.82_Al_0.18_As superlattice buffer. However, the surface subsequently roughened, which may be explained by the other strain relaxation mechanism known as surface undulation.

## Figures and Tables

**Table 1:** Variation of the root-mean-square (RMS) roughness, height difference, the full width at half maximum (FWHM), and dislocation density of the four samples.

Samples	RMS (nm)	Height Difference (nm)	FWHM (Degree)	N_dis_ (cm^−2^)
A	26.7	80.4	0.30256	1.73 × 10^9^
B	24.0	67.1	0.28729	1.56 × 10^9^
C	15.4	48.6	0.25032	1.18 × 10^9^
D	20.5	61.2	0.23887	1.08 × 10^9^
